# The Gene Cluster *Cj0423*–*Cj0425* Negatively Regulates Biofilm Formation in *Campylobacter jejuni*

**DOI:** 10.3390/ijms252212116

**Published:** 2024-11-12

**Authors:** Zhi Wang, Yuwei Wu, Ming Liu, Ling Chen, Kaishan Xiao, Zhenying Huang, Yibing Zhao, Huixian Wang, Yu Ding, Xiuhua Lin, Jiahui Zeng, Feiting Peng, Jumei Zhang, Juan Wang, Qingping Wu

**Affiliations:** 1 School of Bioscience and Bioengineering, South China University of Technology, Guangzhou 510641, China; wz1282074436@163.com (Z.W.); a16620433471@163.com (M.L.); 2 National Health Commission Science and Technology Innovation Platform for Nutrition and Safety of Microbial Food, Guangdong Provincial Key Laboratory of Microbial Safety and Health, State Key Laboratory of Applied Microbiology Southern China, Institute of Microbiology, Guangdong Academy of Sciences, Guangzhou 510070, China; 13503036950@163.com (Y.W.); chenling@gdim.cn (L.C.); 13413422549@163.com (K.X.); scau_zhaoyibing@126.com (Y.Z.); wanghuixian@gdim.cn (H.W.); linxiuhua@gdim.cn (X.L.); zengjiahui@gdim.cn (J.Z.); pengfeiting@gdim.cn (F.P.); zhangjm926@126.com (J.Z.); 3 College of Food Science, South China Agricultural University, Guangzhou 510432, China; 4 Department of Food Science and Technology, Institute of Food Safety and Nutrition, Jinan University, Guangzhou 510632, China; dingyu@jnu.edu.cn

**Keywords:** *Campylobacter jejuni*, biofilm, *Cj0423*–*Cj0425*, pan-genome analysis, pull-down

## Abstract

*Campylobacter jejuni* (*C. jejuni*) is a zoonotic foodborne pathogen that is widely distributed worldwide. Its optimal growth environment is microaerophilic conditions (5% O_2_, 10% CO_2_), but it can spread widely in the atmospheric environment. Biofilms are thought to play an important role in this process. However, there are currently relatively few research works on the regulatory mechanisms of *C. jejuni* biofilm formation. In this study, a pan-genome analysis, combined with the analysis of biofilm phenotypic information, revealed that the gene cluster *Cj0423*–*Cj0425* is associated with the negative regulation of biofilm formation in *C. jejuni.* Through gene knockout experiments, it was observed that the *Cj0423*–*Cj0425* mutant strain significantly increased biofilm formation and enhanced flagella formation. Furthermore, pull-down assay revealed that Cj0424 interacts with 93 proteins involved in pathways such as fatty acid synthesis and amino acid metabolism, and it also contains the quorum sensing-related gene *luxS.* This suggests that *Cj0423*–*Cj0425* affects fatty acid synthesis and amino acid metabolism, influencing quorum sensing and strain motility, ultimately inhibiting biofilm formation.

## 1. Introduction

*Campylobacter jejuni* (*C. jejuni*) is a common foodborne pathogen that can cause diarrhea, gastroenteritis [1], and, in severe cases, Guillain–Barré syndrome [2,3,4]. It is widely distributed globally, with reported cases in the European Union reaching up to 120,946 per year, surpassing those of *Salmonella* [5]. *C. jejuni* is a microaerophilic bacterium that typically cannot thrive in a normal atmospheric environment but can be found in the environment, animals, and food sources [6,7]. Due to their specific structure, biofilms are considered to play a huge role in the tolerance of *C. jejuni* to adverse environments [8,9,10].

Biofilm is formed by microbial cells encased within an extracellular matrix that adhere to various surfaces, allowing the microbes to adapt to changing environments during growth [11]. Over 90% of microorganisms exist in a biofilm form in nature, with 80% of bacterial infections being biofilm-related [12,13]. The transition from planktonic bacteria to biofilm entails significant alterations in structure, gene expression, and response to the environment [10]. Biofilm formation provides a protective mechanism that enhances bacterial tolerance to harsh conditions [14]. *C. jejuni* is capable of forming biofilms as well, utilizing the unique structural characteristics of biofilms to shield itself from unfavorable surroundings.

There are relatively few studies on the biofilm formation mechanism of *C. jejuni*, particularly regarding its regulatory mechanism. In other bacterial species, biofilm formation is commonly regulated through quorum sensing, where changes in cell density lead to alterations in AI-2 concentration, subsequently influencing downstream genes such as LuxR [15,16]. This cascade of events impacts motility, extracellular polysaccharides, and proteins, ultimately affecting biofilm formation [17]. In *Campylobacter jejuni*, LuxS produces AI-2 molecules, but AI-2 is only a byproduct and does not participate in quorum sensing. Although AI-2 levels do not directly indicate quorum sensing activity, they indirectly reflect *luxS* gene expression and thus provide insight into quorum sensing regulation [18]. The transition of pathogenic bacteria lifestyles is also regulated by c-di-GMP concentrations [19]. Low c-di-GMP levels can enhance the expression of cell motility genes, whereas high levels promote the production of cell adhesion factors and increase extracellular matrix components, facilitating bacterial adhesion and biofilm formation [20]. However, only two strains of *C. jejuni* have been reported to respond to c-di-GMP, and c-di-GMP was only detected in DRH212 strains. The presence and role of c-di-GMP in other *C. jejuni* strains are still unclear [21,22].

With the development of whole-genome sequencing technology, a large number of bacterial genomes have been sequenced, making it possible to predict gene functions through methods such as genomics and bioinformatics. Li [23] conducted a pan-genome analysis of *Pseudomonas aeruginosa* and discovered disease-related genes. Her [24] predicted the antimicrobial resistance activity in *E. coli* through pan-genome analysis. It follows that genome-wide association analysis can be used to predict genes associated with biofilm formation. Currently, nearly 800,000 strains of *C. jejuni* have been sequenced, of which about 300 have complete genomes. Bioinformatics analysis can also be used to mine genes related to the biofilm formation of *C. jejuni*. Through pan-genome analysis, it was found that the gene cluster *Cj0423*–*Cj0425* does not exist in all *C. jejuni*. These three genes are hypothetical proteins whose specific functions are unknown. Current research mainly uses omics to discover changes in gene expression. The expression of this gene cluster increased significantly after infection with *C. jejuni* by phage 12673 [25]. The expression of *Cj0425* is upregulated after erythromycin treatment [26]. However, its relationship with biofilm formation is unknown. Therefore, the PCR method was used to amplify the *Cj0423*–*Cj0425* genes in 62 strains of *C. jejuni* in our laboratory, and correlation analysis was performed based on phenotype. It was found that it was negatively correlated with biofilm formation. This study focuses on the impact of *Cj0423*–*Cj0425* on its biofilm and explores its regulatory mechanisms.

## 2. Results

### 2.1. Pan-Genome Analysis Reveals That Cj0423–Cj0425 Is Negatively Associated with Biofilm Formation

**Figure 1:**
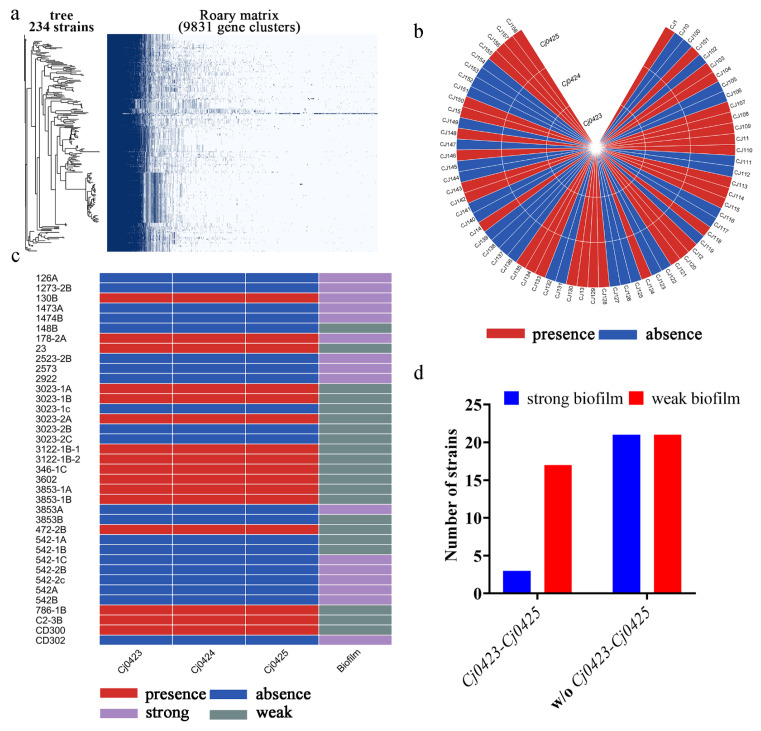
Distribution of *Cj0423*–*Cj0425* in *C. jejuni* and its relationship with biofilm. (**a**) Pan-genome analysis of 234 *C. jejuni* genomes in the NCBI genome database; (**b**) *Cj0423*–*Cj0425* is not present in all *C. jejuni*; (**c**) Association analysis between *Cj0423*–*Cj0425* and biofilm formation; most of the strong biofilm formation strains do not contain *Cj0423*–*Cj0425*; red color indicates the presence of this gene, blue color indicates that the gene is absent, mauve color represents strong biofilm formation ability strain, gray-green color represents weak biofilm formation ability strain; (**d**) Distribution of *Cj0423*–*Cj0425* and biofilm forming ability.

Two hundred and thirty-four complete genomes (Supplementary Table S1) of *C. jejuni* obtained from the NCBI genome database were utilized for pan-genome analysis using Roary [27,28,29,30,31,32,33]. In short, the downloaded FASTA files were annotated using Prokka [32] with default parameters. The annotated GFF files were extracted, and pan-genome analysis was performed using Roary, with all parameters set to default except for the -i parameter, which was set to 85. The analysis revealed a total of 9831 genes, with a core gene number of 856 (Supplementary Table S2) (Figure 1a). It was noted during the analysis that the gene cluster *Cj0423*–*Cj0425* frequently co-occurred. Among the 234 strains, genes *Cj0423*, *Cj0424*, and *Cj0425* appeared at the same frequency, simultaneously appearing 89 times (Figure 1b, figure made by TBtools, Supplementary Table S2) [34]. While these genes are commonly reported as a cluster, their specific functions remain unknown. To investigate further, the distribution of the *Cj0423*–*Cj0425* gene cluster in *C. jejuni* isolated from retail food [7] was examined, showing consistency with whole-genome sequencing results (Figure 1c). Interestingly, strains containing the *Cj0423*–*Cj0425* gene cluster exhibited a weak ability to form biofilms, with 84.21% (16/19) of such strains either unable to form biofilms or forming weak biofilms (Supplementary Table S3). This association between the gene cluster and biofilm formation was found to be statistically significant (*p* = 0.0089, Chi-square). Conversely, strains lacking *Cj0423*–*Cj0425* did not show a significant difference in biofilm formation ability (Figure 1d).

### 2.2. Cj0423–Cj0425 Negatively Regulates Biofilm Formation

**Figure 2:**
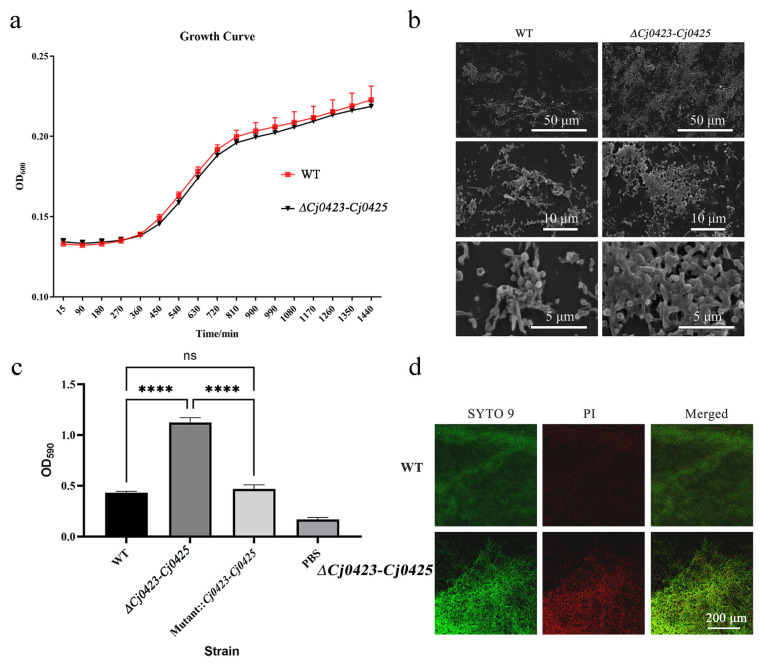
*Cj0423*–*Cj0425* negatively regulates biofilm formation. (**a**) Determining the growth curves of the wild strain and knockout strain by shaking culture under microaerophilic conditions. (**b**) Scanning Electron Microscope observation of biofilm. (**c**) Crystal violet method to determine its biofilm formation ability. (**d**) Observation of biofilm under laser confocal microscope. SYTO-9 is green fluorescence and stains live cells, while PI is red fluorescence and stains dead cells. “ns” means the *p* value is greater than 0.05; “****” means the *p* value is less than 0.0001.

The functions of *Cj0423*–*Cj0425*, particularly related to biofilm formation, remain unknown. *Cj0423*–*Cj0425* is annotated as membrane integral proteins, acidic periplasmic proteins, and periplasmic proteins. Despite the lack of identified highly similar homologous proteins, the exact function of this gene cluster remains elusive. To investigate the relationship between the gene cluster and biofilm formation, gene knockout experiments were conducted, and the growth curves and biofilm formation abilities of different strains were determined. Results showed that, under microaerophilic conditions in MH broth, the growth curve of the knockout strain (*∆Cj0423*–*Cj0425*) did not significantly differ from the wild-type strain, indicating that the *Cj0423*–*Cj0425* gene cluster is non-essential for growth (Figure 2a). This further confirms that the gene cluster is non-core and does not impact the strain’s survival. Subsequently, the biofilm-forming ability was assessed. Quantitative detection using crystal violet staining showed that *∆Cj0423*–*Cj0425* had a significantly stronger biofilm formation ability compared to the wild-type strain, being twice as much as the wild-type strain. (Figure 2c). In other words, biofilm formation is significantly inhibited by *Cj0423*–*Cj0425*. Confocal Laser Scanning Microscopy (CLSM) was used to examine the structure of biofilm, revealing that *∆Cj0423*–*Cj0425* had denser biofilm with higher green and red fluorescence compared to the wild-type strain, indicating increased biofilm formation (Figure 2d). Scanning Electron Microscopy (SEM) further showed that the wild-type strain had loosely distributed biofilm with minimal cell adhesion, while *∆Cj0423*–*Cj0425* had tightly packed cells surrounded by a substantial extracellular matrix, forming a dense biofilm (Figure 2b). These results suggest that *Cj0423*–*Cj0425* may inhibit biofilm formation by regulating extracellular matrix formation.

### 2.3. Cj0423–Cj0425 Reduce the Mobility of C. jejuni

**Figure 3:**
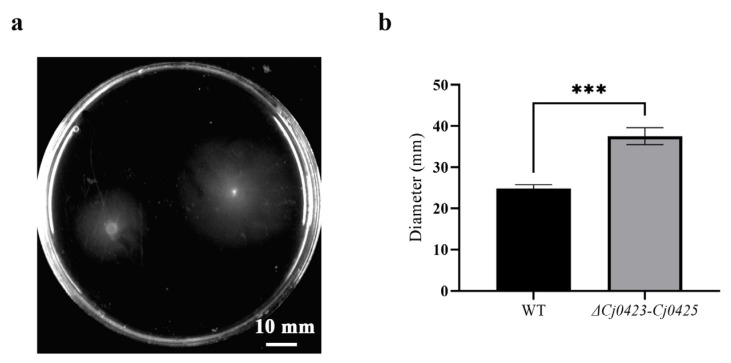
*Cj0423*–*Cj0425* inhibits the mobility of *C. jejuni*. (**a**) Wild-type strain on the left, mutant strain on the right. (**b**) The diameter of the mobility was measured, and the significance was analyzed using *t*-test. “***” means the *p* value is less than 0.0001.

**Figure 4:**
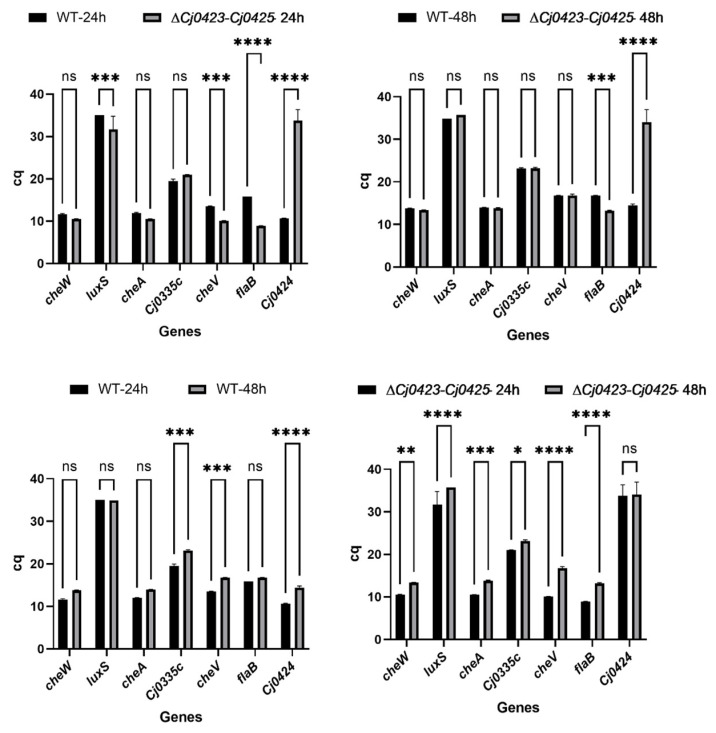
RT-qPCR verifies the results of pull-down, extracts DNA from biofilms at different times, and verifies related genes such as motility, chemotaxis, and quorum sensing. “ns” means the *p* value is greater than 0.05; “*” means the *p* value is less than 0.05; “**” means the *p* value is less than 0.01; “***” means the *p* value is less than 0.001; “****” means the *p* value is less than 0.0001.

Biofilm formation is not only influenced by its extracellular matrix, but also by the impact of mobility on the process. In the initial stage of biofilm formation, the movement of strains facilitates their rapid contact with the medium surface and the development of microcolonies, thereby promoting biofilm formation. The effect of *Cj0423*–*Cj0425* on motility was examined using soft agar (Figure 3). Increased motility was observed in the mutant strains. This phenomenon was further confirmed through RT-qPCR, which revealed a significantly higher expression level of *flaB* in the mutant strain compared to the wild-type strain (Figure 4). These results suggest that the promotion of biofilm formation by *Cj0423*–*Cj0425* is achieved through the enhancement of motility.

### 2.4. Cj0424 Inhibits Biofilm Formation

**Figure 5:**
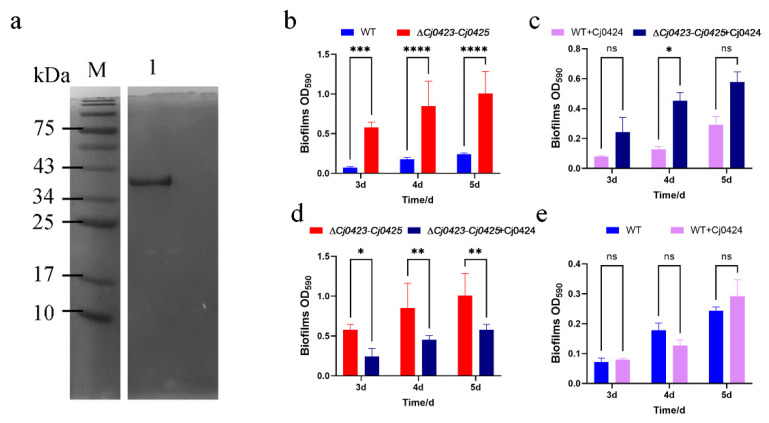
Protein purification and exogenous addition. (**a**) SDS-PAGE of the protein purification, in which Lane 1 is the 500 mM imidazole eluate. (**b**–**e**) The purified protein Cj0424 was added to ∆*Cj0423–Cj0425* and wild-type strain for culture, and the amount of biofilm formation at different times was measured. “ns” means the *p* value is greater than 0.05; “*” means the *p* value is less than 0.05; “**” means the *p* value is less than 0.01; “***” means the *p* value is less than 0.001; “****” means the *p* value is less than 0.0001.

It was observed that biofilm formation and cell motility, as well as extracellular matrix secretion, were significantly influenced by *Cj0423*–*Cj0425*. However, the specific mechanism through which it affects biofilm formation remains unknown. Upon analyzing the domains of these three proteins, it was discovered that Cj0424 contains the MORN (Membrane Occupation and Recognition Nexus) variant repeat domain. Previous study suggests that the MORN domain can act as a protein-binding module to regulate gene function [35]. Therefore, an investigation was conducted to determine whether Cj0424 could directly impact biofilm formation through exogenous addition. Cj0424 was expressed through pET28a plasmid in *E. coli*. As shown in Figure 5a, Cj0424 protein was successfully expressed and purified. Wild-type strain and mutant strain were co-cultured with protein Cj0424 (0.2 μg/L), and the biofilm formation ability at different time points was measured (Figure 5b–e). The addition of Cj0424 significantly inhibited biofilm formation in the *∆Cj0423*–*Cj0425* mutant for 3 to 5 days. While biofilm formation in the wild-type strain was also inhibited, the effect was not significantly different.

### 2.5. Pull-Down Screening for Interacting Proteins in Cj0424

**Figure 6:**
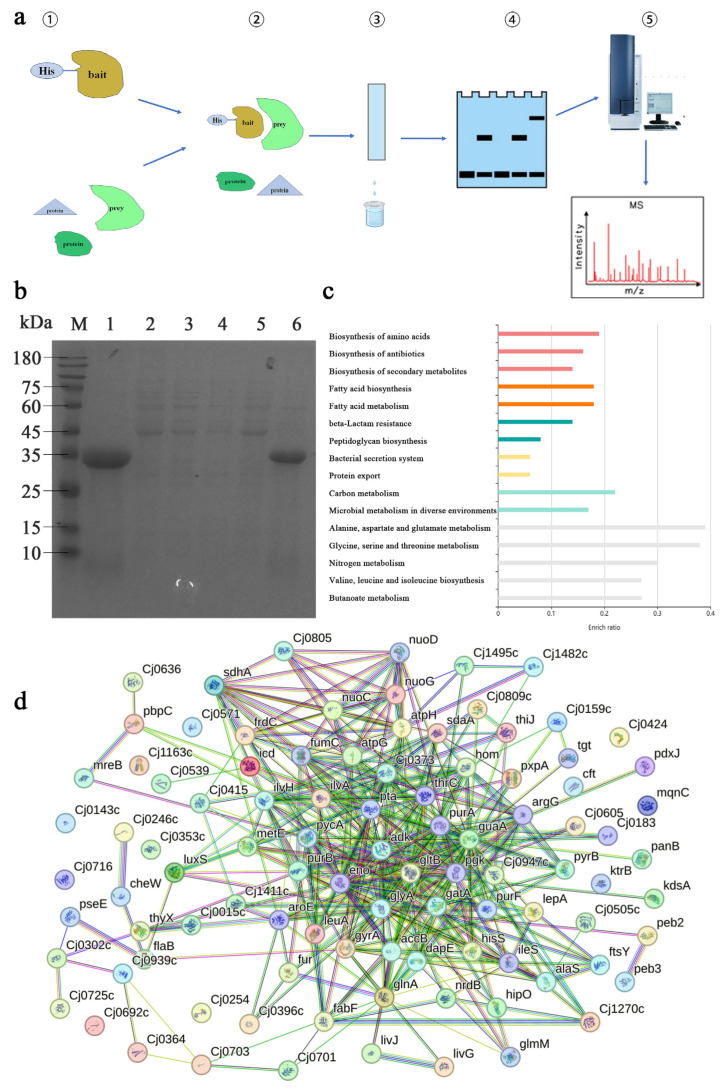
Pull-down identification of interacting proteins: (**a**) Experimental flow chart for pull-down, including ① the bait protein and whole bacterial protein, ② interaction between the bait protein and whole bacterial protein, ③ use of a nickel column to remove unbound proteins and elute interacting proteins, ④ electrophoresis identification of protein interaction results, and ⑤ protein identification using mass spectrometry. (**b**) SDS-PAGE identification of pull-down results, where Lane 1 represents protein Cj0424, Lane 2 is *C. jejuni* whole bacterial protein, Lanes 3–5 depict the impurity washing process, and Lane 6 represents the eluate containing Cj0424-interacting proteins. (**c**) Enrichment of protein pathways. (**d**) Construction of a protein interaction map.

Cj0424 can inhibit biofilm formation, but its function, particularly the proteins with which it interacts to regulate biofilm formation, remains unknown. Here, the pull-down assay was employed to discover proteins that interact with Cj0424, constructing an interaction network (Figure 6a). Purified Cj0424 was utilized to incubate with the whole bacterial protein content of *C. jejuni.* Some non-Cj0424 bands were observed in the pull-down sample (SDS-PAGE, Lane 6), indicating that Cj0424 could pull out a portion of *Campylobacter* proteins (Figure 6b). Mass spectrometry was employed to identify these proteins, resulting in a total of 93 proteins (Supplementary Table S4). Following pathway enrichment, it was observed that these proteins are primarily distributed across pathways such as amino acid synthesis, secondary metabolite synthesis, fatty acid synthesis, and the bacterial secretion system (Figure 6c). The regulation of biofilm formation by extracellular secretions and signaling molecules is highlighted by the enrichment of pathways. Additionally, an interaction was identified between LuxS and Cj0424, suggesting that Cj0424 may mediate quorum sensing and, consequently, impact biofilm formation. String (https://string-db.org/) was used to construct an interaction network for these interactions (Figure 6d). It can be observed that most of the enriched proteins can interact with each other, once again demonstrating the accuracy of the pull-down results.

### 2.6. RT-qPCR Verification of Interacting Proteins

Ninety-two proteins interacting with Cj0424 were identified through pull-down, and the influence of gene clusters on biofilm formation was observed. Genes interacting with Cj0424 were identified using qPCR. LuxS, a quorum sensing-related protein responsible for signal molecule synthesis in other bacteria, was found to have significantly higher expression in ∆*Cj0423*–*Cj0425* than in the wild-type strain at 24 h, with no significant difference at 48 h. This suggests that Cj0424 may influence biofilm formation in the early stages. Simultaneously, an up-regulation in the expression of the chemotaxis-related gene *cheV* was observed at 24 h, and the flagellum-related gene *flaB* showed significant up-regulation at both 24 h and 48 h in ∆*Cj0423*–*Cj0425* (Figure 4). These genes control the strain’s motility, enhance initial colony adhesion, and promote biofilm formation [36]. A comparison between 24 h and 48 h revealed higher expression levels of these genes at 24 h, indicating that the *Cj0423*–*Cj0425* gene cluster predominantly plays a role in the early stages of biofilm formation.

## 3. Discussion

*C. jejuni* is a prevalent foodborne pathogenic bacterium that thrives under microaerobic conditions but can be found ubiquitously in the environment. Despite its growth preferences, it is most frequently reported in Europe [37,38], which is contrary to its growth characteristics. In natural conditions, more than 90% of bacteria exist in biofilms. The structure of a biofilm is dense and has a natural protective layer to protect cells and resist adverse environments. Zhang [39] found that nutrients and external adverse environments are in the form of gradients in the biofilm. They used hydrogen peroxide as environmental pressure and found that, after the bacterial strain formed a biofilm, the hydrogen peroxide could only penetrate the outer layer of the biofilm and the outer cells. A large amount of catalase is synthesized to resist the pressure of hydrogen peroxide and protect internal cells. In fact, *C. jejuni* can resist adverse environments by forming biofilms. Araújo [40] isolated a large number of *C. jejuni* from farms and tested different living environments for these strains, including temperature and oxygen tolerance, and found that, under atmospheric conditions and treatment at 3 °C for a period of time, the strain can still survive through the formation of biofilm. In the early stage, our laboratory [7] also conducted a large amount of food sampling in China and measured the biofilm formation ability of the isolated *C. jejuni*. It was found that nearly 50% of the strains can form biofilms, of which 38.71% can form strong biofilms (Figure 1c).

The biofilm of *C. jejuni* has the function of protecting bacteria, but the mechanism of biofilm formation is still unknown. With the massive sequencing of genomes, it has become feasible to use bioinformatics methods to predict gene functions. A total of 234 complete genomes of *C. jejuni* were downloaded from the NCBI genome database, and pan-genome analysis was conducted on them. It was observed that the gene cluster *Cj0423–Cj0425* is present in only 101 strains, and the majority of strains do not include this gene. At present, there are only a few reports about *Cj0423*–*Cj0425*, but none of them are related to biofilm formation. After adding erythromycin [26,41] and being invaded by phage [25], *Cj0423*–*Cj0425* will significantly increase its expression, but its specific function is still unknown. A PCR amplification of the strains isolated in our lab was performed, combined with the biofilm phenotype information of the strains, revealing a negative correlation between biofilm formation and *Cj0423*–*Cj0425*.

Biofilm is mainly composed of bacteria themselves and their extracellular matrix. Its formation process includes initial attachment, growth, maturity, and other stages [42]. Here, the function of *Cj0423*–*Cj0425* was verified through gene knockout, and it was observed that the biofilm formation ability of the mutant strain was significantly higher than that of the wild-type strain. Combining the results of CLSM and Scanning Electron Microscopy, it was found that the extracellular matrix of the knockout strain was significantly higher than that of the wild-type strain. Compared with wild-type strains, its structure is dense, which is consistent with the composition of biofilm.

**Figure 7:**
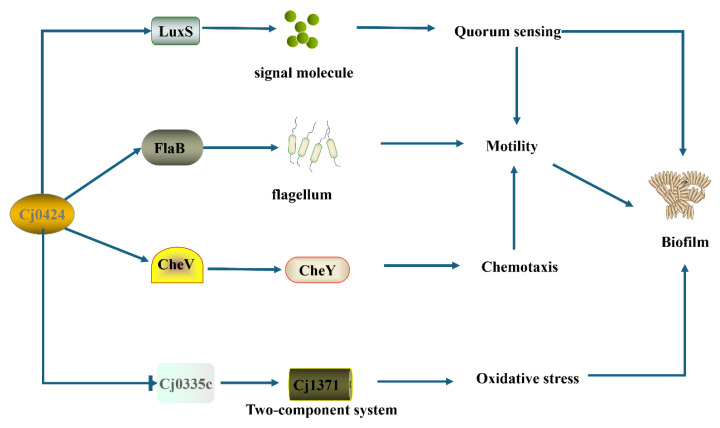
Cj0424 affects biofilm formation through multiple pathways. Cj0424 can regulate biofilm formation through quorum sensing, chemotaxis, motility, and oxidative stress.

The pull-down assay, a commonly utilized technique for screening interacting proteins, was employed by Laventie [43] to investigate the interacting proteins of the signaling molecule c-di-GMP, resulting in the identification of both known and unknown proteins within the c-di-GMP network. Laventie successfully identified 74% of the predicted proteins. Similarly, Jiang [44] utilized the pull-down method to screen the interacting proteins of the uncharacterized functional gene *vp0610* in *Vibrio parahaemolyticus*, identifying 180 interacting proteins, including Hfq, VP0710, VP0793, and CyaA, all of which are known to play a role in biofilm formation. This demonstrates the efficacy of the method, as evidenced by the identification of 93 proteins using the pull-down method. Among these proteins are a significant number known to be associated with biofilm formation, including LuxS, a key component in the bacterial quorum sensing pathway. The LuxS/AI-2 mediated quorum sensing system is prevalent in both Gram-positive and Gram-negative bacteria, with AI-2 serving as a universal signaling molecule across different species [45,46]. The synthesis of AI-2 requires the catalysis of S-ribosylhomocysteine lyase encoded by the *luxS* gene. Bacteria secrete AI-2 into the extracellular environment, and, as cells divide and proliferate, the extracellular AI-2 concentration increases. By detecting these AI-2 signal molecules, bacteria are able to effectively “count” neighboring cells. Once the concentration of AI-2 reaches a critical threshold, this sensing mechanism activates the expression of quorum sensing (QS)-related genes, thereby influencing processes such as biofilm formation and virulence. Through qPCR analysis, it was confirmed that Cj0424 interacts with LuxS, with the mutant strain showing significantly higher *luxS* expression levels compared to the wild-type strain, thus promoting biofilm formation (Figure 7). Additionally, the flagellum-related protein FlaB was identified [47,48], and RT-qPCR results indicated significantly higher expression levels of FlaB in the mutant strain compared to the wild-type strain. This increase in flagella expression likely enhanced strain movement, benefiting the strain. Contact with the media surface resulted in biofilm formation, as confirmed by motility experiments.

An analysis of the pull-down results revealed that the pathways involved include fatty acid biosynthesis, peptidoglycan biosynthesis, bacterial secretion system, and microbial metabolism in diverse environments. These results are also related to the formation of biofilms. Wang [49] studied planktonic and different cellular fatty acid patterns and gene expression of *Listeria monocytogenes* in the biofilm state. It was found that, in the biofilm state, cells synthesize more unsaturated fatty acids and straight fatty acids. This indicates that fatty acids are not involved in the formation of biofilms, but rather changes in the fatty acid profile of cell membranes may affect the formation of biofilms (promoting autoaggregation or surface–cell interactions, e.g., by modifying the hydrophobicity of cell membranes). This study predicted that Cj0424 is a periplasmic protein through bioinformatics and proved through pull-down that it is related to the biosynthesis of peptidoglycan. Peptidoglycan is a component of the cell wall of *C. jejuni*. Frirdich [50] knocked out the peptidoglycan-related gene *pgp2* to affect the morphology of *C. jejuni* and change its intestinal colonization ability. The bacterial secretion system is also enriched in the pull-down results. The extracellular matrix of biofilms is formed by cell lysis or cell exocytosis. At the same time, small molecule metabolites are also secreted out, further regulating the formation of biofilms. This shows that the *Cj0423*–*Cj0425* gene cluster can change the formation of biofilm by affecting the cell morphology, signal molecule transmission, and environmental adaptation of *C. jejuni*. Of course, there are many other enriched proteins, and we will further verify them in subsequent studies.

## 4. Material and Methods

### 4.1. Bacterial Strains and Growth Conditions

*C. jejuni* 3853-1B, isolated from China and cultured in blood plate or MH broth at 42 °C, was purchased from the Guangdong Huankai Biology Sci & Tech. Co., Ltd. (Guangzhou, China). *Escherichia coli* strain DH-5α and Rosetta were preserved in our laboratory. Both *E. coli* were cultured by LB broth (Guangdong Huankai Biology Sci & Tech. Co., Ltd. Guangzhou, China).

### 4.2. Construction of an Cj0423–Cj0425 Mutant and Complemented Strain

All bacterial strains and plasmids constructed and used in experiments are listed in Table 1. All primers used for strains and plasmid construction in this study are shown in Supplementary Table S5. The *Cj0423*–*Cj0425* gene cluster was inactivated by homologous gene recombination in *C. jejuni* 3853-1B. For this purpose, *Cj0423*–*Cj0425* and its flanking sequences were cloned into pM19-T by T-A clone (Takara Biomedical Technology (Beijing) Co., Ltd., Beijing, China), and it was confirmed by sequencing that there was no mutation. Then, inverse PCR amplification was performed from both sides of *Cj0423* and *Cj0425*, and the amplified product and the erythromycin gene fragment were ligated by T4 DNA ligase (Takara Biomedical Technology (Beijing) Co., Ltd.) and heat shock transformed into *E. coli* DH-5α, with erythromycin used to screen positive clones. This plasmid was electroporated into 3853-1B, as in a previous study [51]. The electroporation conditions were 2500 V for 5 ms. Genetic mutants were selected on agar plates containing erythromycin and the correct insertion of erythromycin gene was confirmed by PCR and sequencing. The genetic complementation of the *∆Cj0423*–*Cj0425* mutant strain was performed as described previously [52]. *ΔCj0423*-*Cj0425* was cultivated on blood plates, resuspended in PBS, and adjusted to an OD_600_ of 1.0 to serve as recipient cells. Overnight cultures of the donor *E. coli* strain (pRY107-345) and the helper *E. coli* strain (pRK2013) were subcultured into LB broth and grown to an OD_600_ of 1.2. The cells were then mixed in a ratio of 1:1:10 (donor/helper/recipient), spotted onto blood plates, and incubated overnight at 42 °C under microaerophilic conditions. Following incubation, the mixed culture was resuspended in MH broth and plated on MH plates supplemented with colistin B (6.7 μg/mL), trimethoprim (5 μg/mL), and kanamycin (20 μg/mL). The plates were examined for the presence of *C. jejuni* colonies 3–5 days later and confirmed by PCR.

### 4.3. Measurement of Biofilm Formation

The biofilm formation ability of wild-type and Δ*Cj0423*–*Cj0425* were measured as previously, with minor modifications [40]. Briefly, each well of a 96-well microtiter plate was inoculated with 200 μL diluted to OD_600_ = 0.05 with MH broth without shaking at 37 °C in an atmospheric environment for 6 repetitions. After planktonic cells were removed, the 96-well plate was washed with PBS, then fixed with methanol for 15 min and stained with 230 μL 0.1% (*w*/*v*) crystal violet for 15 min. Then, 230 μL of 33% acetic acid was used to dissolve crystal violet, and OD_590_ was measured, which represents the amount of biofilm formation. The strength of biofilm formation was assessed using OD_590_ measurements compared to a blank control (OD_590_ < 2OD_590-blank_: weak or no biofilm; OD_590_ > 3OD_590-blank_: strong biofilm; 2OD_590-blank_ < OD_590_ < 3OD_590-blank_: medium).

### 4.4. Measurement of Growth Kinetics

The growth curve was determined according to the previous study with modifications [54]. *C. jejuni* was cultured under microaerobic conditions for about 24 h by blood plate, then resuspended in MH broth, adjusted to OD_600_ = 0.05, and added 200 μL diluted bacterial solution to a 96-well plate. The culture condition of the growth curve analyzer was set to 5% O_2_, 10% CO_2_, and 42 °C with the BioTek Epoch Microplate Spectrophotometer.

### 4.5. Comparison of Strain Motility

The motility ability was measured as previously, with minor modifications [55]. All the strains were cultured under microaerobic conditions for about 24 h, and then resuspended in MH broth, adjusted to OD_600_ = 1.0, and added (as a 1 μL resuspension) to the MH ager (0.5%), where they were cultured for about 24 h. Then, we compared the sizes of colony diameters.

### 4.6. Confocal Laser Scanning Microscope (CLSM) Analysis

*C. jejuni* was resuspended from blood plate after being cultured microaerobically, and 500 μL resuspension (OD_600_ = 0.05) was added to a 24-well plate, after which we added a cell slide into the wells. After incubation, the cells were stained using a live/dead cell dye (SYTO-9/PI, Thermo Fisher Scientific Inc., Waltham, MA, USA) and observed under a Confocal Laser Scanning Microscope.

### 4.7. Pull-Down Assay

The pull-down experiment was slightly modified based on the previous method [56]. pET28-B2M was used to construct the expression vector of Cj0424, and *Cj0424* was amplified by PCR, ligated to pET28-B2M by restriction endonuclease and T4 ligase, and transformed into *E. coli* Rosetta cells by heat shock transformation. Cj0424 expression was induced using 0.5 mM IPTG for about 12 h at 28 °C and purified through a nickel column by an AKTA pure system. The purified Cj0424 interacted with the whole bacterial protein of *C. jejuni* and was purified by nickel column, then eluted by imidazole; the eluate was identified by mass spectrometry.

### 4.8. RT-qPCR for Biofilm-Related Genes in C. jejuni

RNAiso plus was used to extract the RNA of *C. jejuni*, and PrimeScript™ RT reagent Kit with gDNA Eraser (Takara Biomedical Technology (Beijing) Co., Ltd.) was used to remove residual genome DNA and synthesize cDNA. TB Green was used for qPCR experiments, and LightCyler 96 (F. Hoffmann-La Roche Ltd., Basel, Switzerland) was used to determine the cq value; *16S rRNA* was used as a control [57].

### 4.9. Statistical Analysis and Software

All the experiments were performed in at least three biological replicates. All statistical analyses were performed using GraphPad, and the specific analysis methods are presented in the results. The genome annotation software was Prokka (version = 1.14.15) [32], the pan-genome analysis software was Roary (version = 3.13.0), and the genome-wide association analysis software was Scoary (version = 1.6.16) [27,28,29,30,31,32,33]. Unless otherwise specified, the default parameters were used.

## 5. Conclusions

Through bioinformatics analysis, we predicted that the gene cluster *Cj0423–Cj0425* plays a negative role in regulating biofilm formation. This prediction was then confirmed using crystal violet staining, SEM, and CLSM. The mutant strain exhibited a significantly higher biofilm formation ability compared to the wild-type strain, with a dense extracellular matrix enveloping the cells. Additionally, the mutant strain showed elevated expression of motility and chemotaxis genes, resulting in increased motility and enhanced attachment to the medium surface for initial biofilm formation. Pull-down assay revealed 93 proteins that interact with Cj0424, including quorum sensing-related protein LuxS, chemotaxis-related protein CheV, and motility-related protein FlaB. Enriched pathways such as fatty acid biosynthesis and bacterial secretion system were identified to influence biofilm formation. In conclusion, *Cj0423*–*Cj0425* negatively regulates biofilm formation in *C. jejuni* by impacting motility, quorum sensing, extracellular secretion, and other related pathways.

## Figures and Tables

**Table 1:** Strain and plasmid information.

Strain or Plasmid	Characteristic	Source or Reference
***Campylobacter jejuni***		
3853-1B	Weak biofilm formation	Our lab
3853-1BΔ*Cj0423–Cj0425*	*ΔCj0423–Cj0425*	This study
3853-1BΔ*Cj0423–Cj0425:345*	Complementation strain	This study
***Escherichia coli***		
DH-5α		Our lab
Rosetta		Our lab
**Plasmids**		
pMD19-T		Takara Co., Ltd., Beijing, China
pRY107	Shuttle vector	[53]
pCj345	Suicide vector	This study
pRY107-345	pRY107-Cj0423–Cj0425	This study
pET28a-B2M	Expression vector	Our lab
pET28a-Cj0424	Expression Cj0424	This study

## Data Availability

During the revision of this work, the authors used ChatGPT to edit the language to enhance the readability. After using this service, the authors reviewed and edited the content as needed and take full responsibility for the content of the publication.

## Supplementary Materials

The following supporting information can be downloaded at: https://www.mdpi.com/article/10.3390/ijms252212116/s1.
