# Supplementary material for: The Gene Cluster Cj0423–Cj0425 Negatively Regulates Biofilm Formation in Campylobacter jejuni
Source: Int J Mol Sci. 2024 Nov 12;25(22):12116. doi: 10.3390/ijms252212116 (PMC11595200; doi:10.3390/ijms252212116)
Supplement: Supplementary file 1 [file ijms-25-12116-s001.zip › Supplementary Table S5.docx]

**Supplementary Table 1 primer list**

| **Primer** | **Sequence(5’-3’)** |
| --- | --- |
| Cj0423_F | TATTCCTGCTCCTTCT |
| Cj0425_R | TTCAGCCGTTATTATCT |
| pCJ_345r_F | ccgCTCGAGCACTTGCACCTTCCATGTAAT |
| pCJ_345r_R | ccgCCATGGTAACTTATATCAATGGAGATTTCCAATAA |
| ermC_F | ccgCTCGAGATGAACGAGAAAAATATAAAAC |
| ermC_R | ccgCCATGGTTACTTATTAAATAATTTATAGCTA |
| Cj0423-*Bam*H I | CCGGGAGCTCCATGGAAGGTGCAAGTGGCG |
| Cj0425-*Xho* I | CCGCTCGAGTTATTGGAAATCTCCATTG |
| pRY107-*Xho* I | CCGCTCGAGGGGGGGCCCGGTACCCAATTC |
| pRY107-*Bam*H I | CCGGGATCCACTAGTTCTAGAGCGGCCGCC |
| Cj0424_R_*Xho* I | ccgCTCGAGCTAATTTTTAGGTTTTG |
| Cj0424_F_*Eco*R I | ccgGAATTCATGACAAAATTTTTAAGCATTT |
| 16S_F | GCTCGTGTCGTGAGATGTTG |
| 16S_R | TCACCGTAGCATGGCTGAT |
